# Semiochemical and Vibrational Cues and Signals Mediating Mate Finding and Courtship in Psylloidea (Hemiptera): A Synthesis

**DOI:** 10.3390/insects5030577

**Published:** 2014-07-21

**Authors:** Umar K. Lubanga, Christelle Guédot, Diana M. Percy, Martin J. Steinbauer

**Affiliations:** 1 Department of Zoology, La Trobe University, Melbourne, Victoria 3086, Australia; E-Mail: M.Steinbauer@latrobe.edu.au; 2 Department of Entomology, University of Wisconsin, Madison, WI 53706, USA; E-Mail: guedot@wisc.edu; 3 Natural History Museum, London, SW7 5BD, UK; E-Mail: d.percy@nhm.ac.uk

**Keywords:** sexual selection, mate selection, mating system, male mating strategy

## Abstract

Mate finding and courtship involve complex interactions that require close coordination between individuals of the opposite gender. Well-organized signalling systems, sometimes involving a combination of signal modalities, are required to convey species-specific and individual information to members of the opposite gender. Previous studies of psyllids have focused on single-signal modalities and have largely ignored the potentially interdependent nature of different types of signals. Several studies have shown that semiochemicals play a role in psyllid mate finding. However, long-range semiochemical sex attractants, such as the highly volatile sex pheromones used by many Lepidoptera (molecular weights <300), are yet to be identified. The compounds identified thus far, namely 13-methylheptacosane (from *Cacopsylla pyricola*) and dodecanoic acid (from *Diaphorina citri*), seem to have short range activity or no activity under field conditions. The possible role played by cuticular hydrocarbons in psyllid courtship remains largely ignored. Conversely, many psyllid species rely on vibrational signals for mate finding and mate assessment during courtship. This apparent disproportional reliance on vibrational rather than semiochemical signals suggests that vibrational signals have been more influential in sexual selection in psyllids. However, male fitness, female choice and benefits accrued from selecting fitter males remain poorly understood.

## 1. Signals and Strategies of Insect Sexual Selection

Mating behaviours are typically complex and comprise a series of interdependent cascading events [1]. Individuals must search for mates, engage in courtship, copulate and might need to recover from the cumulative costs of prior events. Searching and courtship involve communication and coordination between individuals that may not necessarily share similar intentions [2,3,4]. Sexual conflict arises because males and females have divergent interests linked to gender-based differences in size and the mobility of gametes, *i.e.*, anisogamy [1,5].

Generally, males can afford to produce relatively larger numbers of gametes compared to females, whose gametes are usually larger and more costly to produce [6]. Consequently, males optimise their reproductive fitness by maximising the number of mating events, whereas females optimise fitness by selecting and mating with higher quality males, *i.e.*, females are choosy [5,6,7]. Female choosiness is widely reported in several insect orders [8,9,10]. In some species, males may invest significantly in reproduction, leading to an apparent reversal of sex roles [11]. In such systems, female fitness is often an increasing function of the number of matings, especially when females receive direct benefits (e.g., nuptial gifts, access to food resources defended by males) from males during copulation [11]. For example, virgin and mated female *Pieris protodice* (Lepidoptera: Pieridae) solicit copulations to obtain fresh spermatophores from males [12].

Mating systems, such as polygyny (males mate with several females), polyandry (females mate with several males) and monoandry (an exclusive association with a single member of the opposite sex) have been reported in several insect orders, such as Orthoptera, Diptera and Hemiptera [13,14,15]. Similarly, male mating strategies, including female defence (males defend one or more females), *i.e.*, male dominance and competition for choosy mates, resource defence (males defend resources critical to female survival) and self-advertising or leks (males position themselves around locations frequented by females) are widely reported among various insect orders [16,17,18]. Mating systems and male mating strategies determine sex roles (which sex searches and which sex signals), the efficacy of mate finding and the frequency of courtship [1,18].

Insects rely on a wide range of signalling modalities to communicate [19]. Orders, such as Lepidoptera and Hymenoptera, rely heavily on chemical and visual signals [20,21,22,23]. Orthoptera and some families of Hemiptera, most notably Cicadidae, primarily rely on airborne acoustic signals (e.g., [24,25]). Other hemipteran families (e.g., Pentatomidae and Cydnidae) are known to utilize substrate borne vibrations for mate location and assessment [26]. Orders in which chemical signalling is dominant usually possess, not surprisingly, highly developed olfactory systems [27], while those that rely on vibrational signals tend to possess well developed sound producing and perception organs [28,29]. The relative degree of complexity of morphological traits associated with signalling usually reflects the relative importance of a signal modality within a species [27,30]. The diversity of signals utilised reflects phylogenetic traits retained over evolutionary time, often as a consequence of physical constraints imposed by their environment [1].

Long-range signals are required to convey species and gender-specific information during searching, while short-range signals convey information about a specific individual, which is important during courtship [4,31]. In the case of chemical signalling, highly volatile semiochemicals (those with molecular weights (MWs) <300) are involved in long-range transmission of information, while heavier compounds (such as cuticular hydrocarbons with MWs >300) are considered more effective for short-range communication [1,32,33]. Single-signal modalities may be capable of conveying information during both mate finding and courtship. However, distortions (due to contamination or background noise (e.g., [34]) and, in some cases, deception from senders have driven some species to rely on more than a single signal modality, *i.e.*, multimodal signalling systems [35]. For example, a multimodal signalling system involving semiochemicals and vibrational signals has been reported in the green vegetable bug, *Nezara viridula* (Hemiptera: Pentatomidae) [36,37]. Furthermore, mating and courtship signals are often under strong stabilising selection pressure to maintain species-specific information [38]. Consequently, species and mate recognition signals are expected to show little intraspecific variation, thus limiting the information available for mate assessment. Species, such as *N. viridula*, address this dilemma by utilizing a multimodal signalling system or, in cases where a single modality is utilized (e.g., vibrational signals), by varying mate finding and courtship signals [36,37]. Variation in mate finding and courtship signal characteristics has also been reported in field crickets and cicadas [39,40].

## 2. Introduction to the Psylloidea

**Figure 1:**
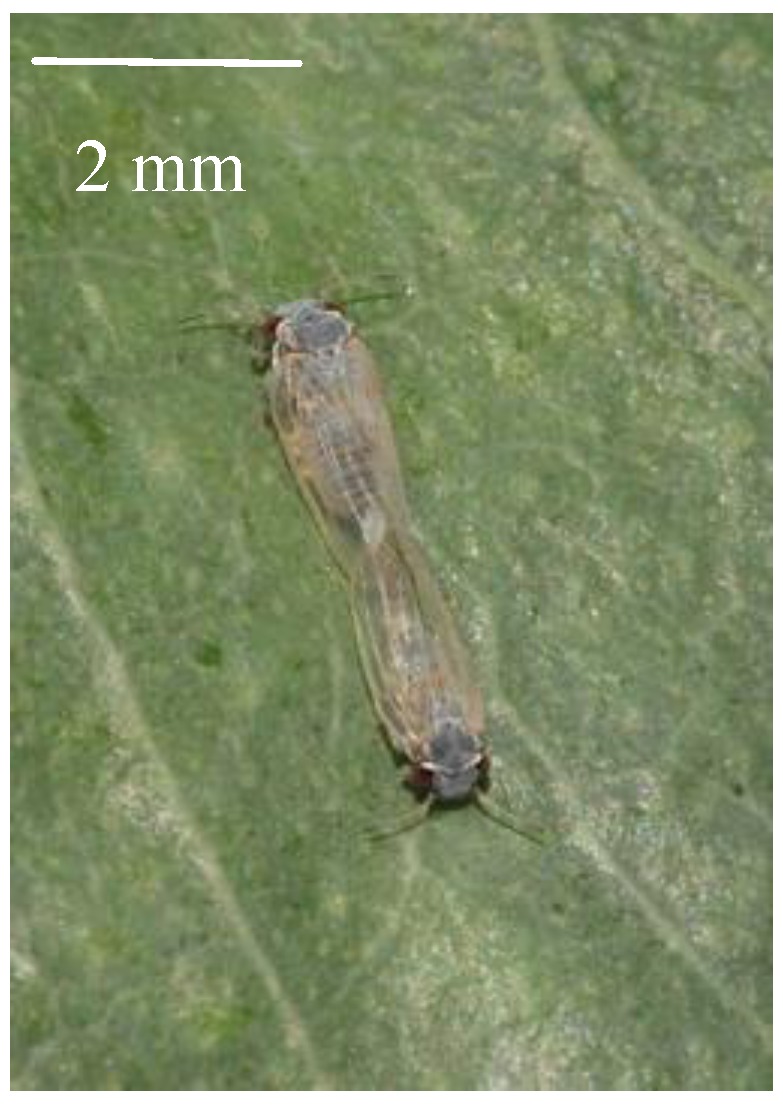
A pair of blue gum psyllids (*Ctenarytaina eucalypti*) *in copula*; female at top, male at bottom. Photograph courtesy of Ben Twist.

Psylloids (psyllids or jumping plant lice) are small, exclusively phytophagous insects (Figure 1). The superfamily, Psylloidea, is highly diverse, comprising over 3800 described species distributed worldwide in all major zoogeographical regions [41,42]. Psyllid feeding can harm host plants by causing leaf chlorosis, necrosis and premature abscission [43,44]. Species, such as *Diaphorina citri* (Liviidae), *Bactericera cockerelli* (Triozidae) and *Cacopsylla picta* (Psyllidae), are economically important, because of their ability to vector harmful plant pathogens [45,46,47,48,49,50]. Psyllids reproduce sexually, and the immature ones pass through five nymphal instars before becoming adults [44]. Nymphal biologies include species that are free-living (nymphs do not develop beneath a formed shelter or in a gall), gall-forming (nymphs induce and develop inside a plant gall), lerp-forming (nymphs develop beneath a shelter of their own making) or, in some cases, inquilines (nymphs may reside beneath shelters or in galls made by other species) [44,51,52]. Seasonal dormancy in the form of either reproductive diapause or an “oligopause” have been reported in species endemic to regions with moderate to severe winters [53,54,55].

## 3. Psyllid Reproductive Biology and Mating Systems

In some species, both genders reach reproductive maturity within 24–48 h post-eclosion [56,57,58]. Oviposition usually commences within 24 h after mating, but may be delayed when females mate within 48 h post eclosion [56,57]. Studies of vibrational communication have shown that only unmated females responded to male signals, implying that there may only be a single copulatory event in some species [59,60]. However, it has been shown that females of some species must mate multiple times, in most cases with different males, to realize their reproductive potential [58,61,62]. *Cacopsylla pyricola* females have a requirement to mate at least twice within ten days, while *Trioza erytreae* (Triozidae) females require at least four matings to continuously produce fertile eggs [58,62]. In addition to increased fertility, females can benefit from increased longevity, as was reported in *C. pyricola* [62]. Following mating, males resume searching, while females may take longer before becoming sexually receptive again [58,59,63,64]. In *B. cockerelli*, the male refractory period lasts less than 24 h, while that of females may last a minimum of 48 h [64].

Greater numbers of receptive males relative to receptive females (male-biased operational sex ratios) usually result from gender-related differences in refractory periods and sets up conditions for male-male competition. In extreme cases, male-male competition may lead to female harassment and reduced oviposition [61,65]. Females of some insects, including psyllids, negate the effects of a biased operational sex ratio by not emitting signals [64,66]. This may explain why females of *B. cockerelli* were unattractive and even repellent to males during the refractory period [64] and may also account for the observation from vibrational studies of only a single mating event for females *versus* multiple events for males [59].

Whether or not females of a particular psyllid species typically mate once or more than once may be linked to the length of time from adult emergence to reproductive maturity and/or receptivity and to protandry (the seasonal emergence of males prior to females) [56,62]. Typically, protandry is more common in temperate [62,67] compared to tropical species [56].

## 4. Male Mating Strategies

**Figure 2:**
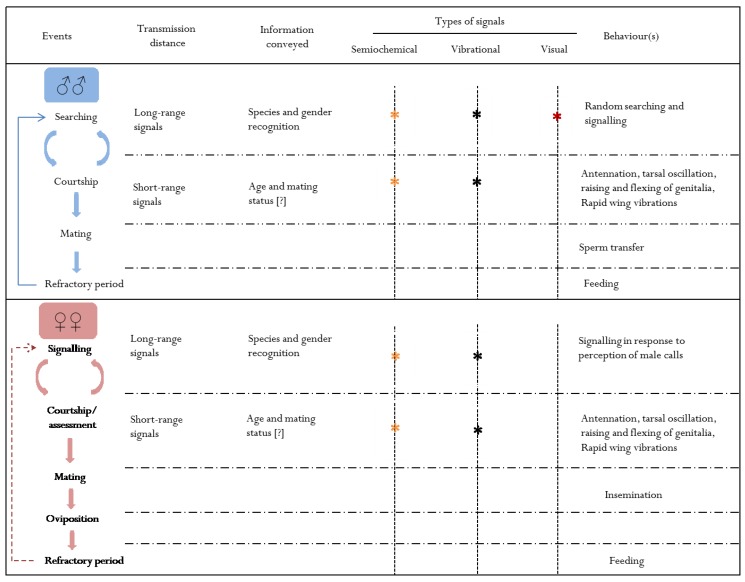
Generalised schema of behaviours from mate location through to mating and the refractory period for male and female psyllids, respectively. Key: * = signal studied, but role and transmission distance remains unclear; * = signal studied and believed to play a role in both mate finding and courtship; * = putative signal; [?] = probable information conveyed. The dotted arrow in the schema for females indicates that some species may mate only once.

Most studies of psyllid mating behaviour provide no distinction between mate finding and courtship events (see Figure 2). Here, we use the term mate finding synonymously with searching to refer to all activities leading to mate location and contact between males and females. Courtship is used to refer to close-range activities between males and females from initial contact to copulation. Generally, mate finding involves random movements by males followed by directed searching once they have perceived a female signal. In contrast, females are sedentary and, in most cases, only signal in response to male signals if receptive to mating [58,59,62,63,67,68,69].

While searching and mate finding may be protracted, courtship is typically brief [68,70] and, in most cases, lacks extravagant behaviours associated with courtship in other insect taxa, e.g., dances, exchange of nuptial gifts and serenades [71,72,73]. The small size of most psyllid species (~2–8 mm) complicates studies of courtship and partly explains why these behaviours are poorly understood. Nevertheless, rapid wing vibration and tarsal oscillation, such as observed in *Cardiaspina densitexta* and *Cacopsylla pyricola* courtship [67,68,70], suggest the use of vibrational signalling during courtship (note: the functional significance of tarsal oscillations to the production of substrate-borne vibrations has not been demonstrated). Antennation of the bodies of individual *B. cockerelli* and *C. pyricola* prior to copulation could suggest a role of cuticular hydrocarbons in mate assessment during courtship [68,70,74].

In most psyllid species, male-male competition is characterized by active searching for females, but not physical combat [58,63,68]. As mentioned, females are often sedentary and solitary or occur in loose aggregations [58,63,67,68]. Female choice seems to play an important role in determining the outcomes of courtship events [58,67,68]. Indeed, in some cases, males are rejected several times before successful mating occurs [67,68]. In some species, such as *A. dobsoni*, *B. cockerelli* and *C. pyricola*, males often attempt to mate with other males or with mating pairs without any obvious aggressive behaviour. These behavioural characteristics suggest that psyllid males maximise their fitness via competition for choosy mates, *i.e.*, scramble competition (see [16]). Males are apparently the “search and signal” sex and females are the “sedentary and signal” sex. Most vibrational studies have found that females do not initiate signalling, but signal only in response to the perception of male signals [59,60,63,75,76]. However, in a few species, and only on rare occasions, females signal in the absence of males [77,78]. Thus, females may attract males using long-range signals (*i.e.*, volatile semiochemicals or vibrational signals), and males may then be assessed by females using short-range signals (cuticular hydrocarbons or vibrational signals) (see Figure 2). Short range, sex-specific signals that are used for mate assessment are therefore expected to vary more in males compared to females. (Note: We propose that for psyllid semiochemical signals, long range should refer to distances greater than the length of a host plant module (e.g., photosynthetic branchlet or leaf), since these signals should be able to attract conspecific insects from neighbouring plants. For vibrational signals, long range should refer to distances greater than the length of a plant module, but limited to the same host, since these signals cannot transmit across open spaces. In contrast, short range for both semiochemical and vibrational signals should mean less than one body length of a conspecific insect.

Mating systems are unlikely to be similar across Psylloidea and male behaviours, such as attempting to mate with other males and with *in copula* pairs, suggesting the potential existence of alternative mating strategies, such as female mimicry and mate guarding, which have been found in other insects, but not considered in psyllids [2,16].

## 5. Semiochemical Signalling in Psyllids

Studies of the psyllid olfactory system have revealed a relatively simplified system. Unlike the complex antennae of insects in taxa, such as Lepidoptera and Hymenoptera, psyllid antennae are typified by sparse antennal sensilla and correspondingly few olfactory glomeruli [79,80,81]. It was only relatively recently that semiochemical signalling was reported to play a role in psyllid mate attraction [82]. Earlier studies had mainly focused on the role of semiochemicals in host plant location and selection [83,84,85]. Nevertheless, males of *C. bidens* (Psyllidae) were shown to be attracted to host plants supporting females, and their antennae produced greater electroantennographic responses to semiochemicals from pear hosts infested with females [82]. Additionally, while investigating the aggregation behaviour by post-diapause winter forms of *C. pyricola*, Horton and Landolt [86] found that males were more abundant on pear shoots currently or previously occupied by females compared to un-infested shoots or shoots previously occupied by males only. From these studies, it was not clear whether the males were more attracted to host plants or to female-produced semiochemicals. Subsequently, Horton *et al.* [87] confirmed that in summer forms of *C. pyricola*, it was female semiochemicals rather than plant semiochemicals that attracted conspecific males. Later, Guédot *et al.* [88] obtained similar results using the winter form of *C. pyricola*, suggesting the potential existence of female semiochemicals attractive to males. The existence of female-produced semiochemicals was confirmed in field trials, which showed that *C. pyricola* males of both types were more attracted to sticky traps baited with live females compared to traps baited with live males or traps that were left unbaited [89].

To-date, males of four psyllid species have been shown to be attracted to female-produced semiochemicals, namely: *C. bidens*, *C. pyricola*, *B. cockerelli* and *D. citri* (Table 1). In contrast, females are usually neither attracted nor repelled by male semiochemicals, with the exception of female *B. cockerelli*, which are repelled by male odour [90]. Although males and females are generally neither attracted nor repelled by same sex odours, cases of female-female repulsion (e.g., *C. pyricola* [91]), male-male attraction (e.g., *B. cockerelli* [90]) and male-male repulsion (e.g., *C. pyricola* [88]) have been reported. These results suggest that semiochemicals are utilized differently in different psyllid species. Furthermore, olfactometer bioassays using *C. pyricola* and *B. cockerelli* showed that whole body extracts of females were equally as attractive to males as live females [90,91].

These findings by Guédot [90] and colleagues suggest that female semiochemicals could be isolated and identified from whole body extracts using a combination of gas chromatography and mass spectrometry (GC-MS) and olfactometer bioassays. (Note: the term “whole body extracts” has been used by some authors synonymously with other terms, such as insect extracts, extracts and cuticular extracts to refer to extracts obtained by soaking whole bodies of freshly killed insects in non-polar organic solvents [90,92]. We propose a more precise application of the terms whole body extracts and cuticular extracts; whole body extracts is used to refer to extracts obtained by soaking or rinsing whole bodies of freshly killed insects. Such extracts may include compounds not typically associated with the insect’s cuticle, e.g., internal lipids and exocrine gland secretions. The term cuticular extracts is used to refer to those extracts obtained exclusively from the insect’s cuticle and likely to be composed entirely of cuticular hydrocarbons (CHCs)).

Male *C. pyricola* were shown to prefer post-diapause females to diapausing females [93]. Subsequently, by comparing the chemical profiles of whole body extracts using GC-MS of diapausing and post-diapause males and females, Guédot *et al.* [94] found that post-diapause females produced significantly larger quantities of 13-methylheptacosane. In olfactometer assays, live females were shown to be as attractive as a comparable quantity of synthetic 13-methylheptacosane, while field trials showed that males were attracted to synthetic 13-methylheptacosane. This provided the first evidence of a female-produced semiochemical capable of attracting male psyllids from neighbouring host plants. Using a similar approach, dodecanoic acid was identified as the female semiochemical attractive to male *D. citri* [92]. However, field trials using traps baited with dodecanoic acid did not increase the total catch of *D. citri* males compared to blank traps [92].

The physical properties of semiochemicals can be used to infer their likely suitability as long-range mate attractants. An effective long-range sex attractant should be highly volatile. The volatility of organic compounds is primarily a function of their molecular weight with an upper weight limit for airborne pheromones of about 300 [95]. With a molecular weight of 395, 13-methylheptacosane is, at best, weakly volatile and, therefore, most likely to function as a short-range attractant. Conversely, dodecanoic acid with a molecular weight of 200 seems sufficiently volatile to be a long-range attractant, although it failed to attract *D. citri* males to traps in the field [92]. Dodecanoic acid has been found in several other insect species, but to date, there is no record of its use as a sex attractant.

**Table 1:** Summary of signals mediating mate finding and courtship in Psylloidea.

Family	Species	Semiochemical (s)	Vibrational Signal	Nature of Vibrational Signal		Mechanism of Vibrational Signal Production	Ref.
				Substrate Borne Vibrations	Air Borne Vibrations		
Aphalaridae	*Anoeconeossa* sp. *Anoeconeossa unicornuta*		●	■		RWV, TO [?]	[96,97,98]
	*Aphalara affinis, Aphalara polygoni*		●	■		RWV	[60,96]
	*Australopsylla* sp*.*		●	■		RWV/TO [?]	[96,97,98]
	*Blastopsylla* sp*.*		●?			TO [?]	[97]
	*Cardiaspina albitextura, C. retator, C. tenuitela, C. densitexta*		●	■	Faint whirring sound	RWV	[67,98]
	*Cardiaspina maniformis, C. fiscella*		●	■		RWV	[96,99]
	*Craspedolepta gloriosa, C. campestrella, C. nervosa, C. flavipennis, C. nebulosa*		●	■		RWV	[60]
	*Creiis* spp*.*		●		High pitched buzzing calls	RWV	[76,98]
	*Ctenarytaina* sp*.*		●?			TO [?]	[97]
	*Glycaspis spp. G. brimblecombei, G. johnsoni, G. neureta*		●	■		RWV/TO [?]	[97,98]
	*Lasiopsylla rotundipennis*		●	■		RWV	[96,98]
	*Spondyliaspis* sp.		●	■		RWV	[96,98]
	*Phellopsylla* sp*.*		●	■		RWV	[96,98]
Carsidaridae	*Protyora sterculiae*		●?			TO [?]	[97]
Liviidae	*Diaphorina citri*	▲ (A)	●	■		RWV	[78,92,100]
	*Eremopsylloides amirabilis*		●			RWV	[63]
	*Livia juncorum*		●		Short buzzing sounds	RWV	[60,101]
	*Pachypsylloides citreus*		●	■		RWV	[63]
Psyllidae	*Cacopsylla bidens*	▲				?	[82]
	*Cacopylla pyri*		●	■		RWV	[77]
	*Cacopsylla pyricola*	▲ (B)				RWV	[88,91,94]
	*Colposcenia aliena*		●	■		RWV	[63]
	*Livilla ulicis*		●	■		RWV	[60,96]
Triozidae	*Aacanthocnema dobsoni*		●	■		RWV	[59]
	*Bactericera cockerelli*	▲				?	[90]
	*Bactericera perrisii, B. kratochvili, B. calcarata, Eryngiofaga deserta*		●	■		RWV	[63]
	*Schedotrioza apicobystra, S. cornuta, S. distorta, S. marginata, S. multitudinea, Schedotrioza* sp.		●	■		RWV	[59]
	*Trioza tricornuta, T. percyae*		●			RWV	[59]
	*Trioza* spp. *Trioza acutipennis, T. nigricornis, Trioza urticae*		●	■	Short buzzing sounds	RWV	[59,63,101]

Key: ▲ = species in which semiochemicals have been found to play a role in mate finding; A = dodecanoic acid; B =13-methylheptacosane; ● = species investigated for use of vibrational signals; ●? = species in which tarsal oscillations have been observed, but no vibrations detected; ■ = species from which vibratory signals have been recorded; RWV = rapid wing vibrations; TO [?] = tarsal oscillations (putative mechanism of vibrational signal production); ? = unknown mechanism of vibrational signal production.

Long-range volatile semiochemicals have previously been collected and identified after excising whole pheromone glands and analysing their chemical contents [102]. However, pheromone glands have not yet been identified in Psylloidea. Cuticular hydrocarbons (CHCs) are a complex blend of n-alkanes, methyl-branched alkanes and alkenes, typically with chain lengths of about 21–37 carbon atoms. If biologically active, these compounds are likely perceived at short range, possibly via contact chemoreception [33,103,104].

Clearly, further work is required to isolate semiochemicals responsible for the behaviours observed during experimental studies. Additionally, the possibility that high MW semiochemicals may have only short-range biological activity has been ignored. Nevertheless, semiochemicals with short-range (contact) activity are believed to be ideal indicators of transitory states relevant to mate quality and assessment, e.g., reproductive status/age and receptivity [4,105,106,107]. Although the ultimate function of cuticular hydrocarbons is to protect insects from desiccation [32], they are well known for their role in intraspecific communication [4,32,33].

## 6. Vibrational Signalling in Psyllids

**Figure 3:**
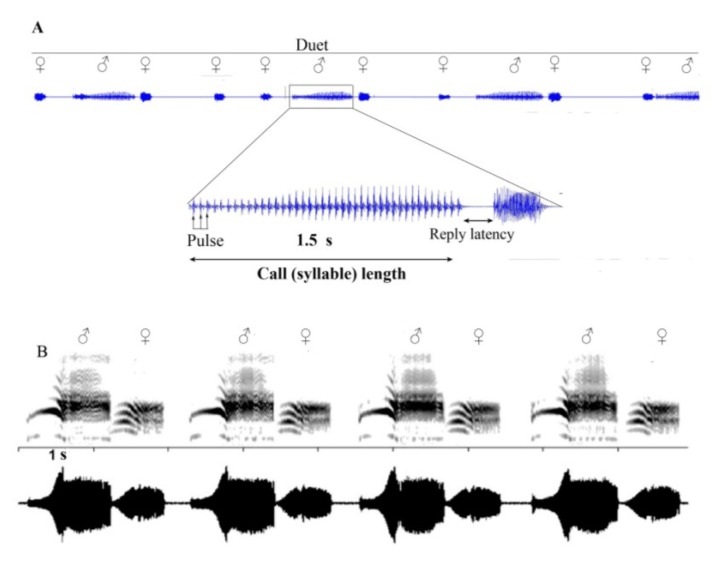
Vibrational duetting in triozid psyllids. (**A**) *Aacanthocnema dobsoni*; long, simple male call (syllable) and short female reply (syllable). (**B**) *Schedotrioza apicobystra* (published with permission from CSIRO publishing) short and complex, tightly synchronized male-female duet. s = seconds.

Psyllids rely on simple stridulatory mechanisms to produce vibratory signals. By vibrating their forewings, a single row of ridges on the anal vein rubs against similar structures on protruding ridges on the meso- and meta-thorax to produce vibrations [76,96,108]. Such wing movements are commonly reported in the literature as wing flicks [63,67,78], but because some types of wing flicks are performed without producing distinct vibrational signals (during wing flexing or stretching), we refer to wing movements that produce signals as rapid wing vibrations. Signal characteristics, such as call (syllable) length, pulse number and reply latency, are used for species and gender recognition (Figure 3) [59,78].

Studies have indicated that temporal variations (e.g., call length, latency period and rate of call) and frequency modulation are more readily detected than structural variation [59,67,77,78]. It may be that subtle temporal and frequency shifts, rather than overall structural variation, distinguish changes in signal information associated with searching (at longer range) or courtship (at shorter range). However, under this scenario, there may still be limited intraspecific signal variation and, therefore, reduced information available to females for effective mate assessment and discrimination. Nevertheless, if signal structure in psyllids is under strong stabilizing pressure to maintain species-specific information, then varying signals via temporal or frequency shifts during mate finding and courtship may be sufficient for effective mate assessment and may be an effective compromise between different selection pressures.

Although evidence for variation in signals associated with mate proximity is limited in Psylloidea, the variation in signal structure that has been reported highlights the potential value of vibrational signals in qualitative mate assessment (sexual selection). If females use vibrational signals to assess and select between males, then there should be a correlation between desirable male traits and specific signal characteristics. More studies are needed to compare signals from solitary males with those of males producing mate finding and courtship signals and at varying distances from receiving females. Wenninger *et al.* [78] reported a significant correlation between fundamental frequency (lowest frequency of a periodic waveform) and male body mass in *D. citri*, which could potentially convey information about male fitness to receiving females.

Selective pressure on signal stabilization to convey species-specific information may vary between species and may be influenced by certain aspects of the species’ ecology. Percy *et al.* [59] showed that divergence in the signal of species sympatric on the same plants varies depending on whether the species are closely or distantly related. It is likely that species that live in sympatry with closely related species are under more signal stabilization pressure compared to species that live in sympatry with distantly related species or without other psyllid species on the same host plants. We currently do not know how species living in contrasting habitats respond to the challenges imposed by either stronger or more relaxed signal stabilization pressure. Percy *et al.* [59] found that species with short, complex signals exhibit less intraspecific variation than species with long, simple signals and that the former tend to engage in more tightly synchronized male-female duets (e.g., *Schedotrioza* Figure 3B). If species with long, simple calls exhibit greater intraspecific variation and less synchronized duetting (e.g., *Aacanthocnema*; Figure 3A), this may suggest weaker stabilizing selection.

Species that have a short male signal with limited intraspecific variation may carry less qualitative information to females, reducing the effectiveness of female choice in mate selection [109]. Under these circumstances, other signal modalities, such as visual signals and semiochemicals, may be important (e.g., [110,111]). Wenninger *et al.* [78] suggested that males rely on vibrational signalling in the absence of olfactory cues, but that they may also, conversely, rely less on vibrational signals in the presence of olfactory cues. This could imply a trade-off in the use of acoustic *versus* olfactory signals, especially where acoustic signals may be associated with greater risk, e.g., attraction of unwanted competition by eavesdropping males or of predation [109]. Percy *et al.* [59] reported longer female reply latencies in species with long simple male calls (e.g., *A. dobsoni*). Increased temporal variation in reply latencies, together with the absence of tightly synchronized duetting, may allow for the transmission of qualitative information. Additionally, female signals generally remain short, even when male signals are longer [59], perhaps because the risk of predation for the more sedentary female is greater, and therefore, variation in the reply latency may be used to transmit information with less risk. Thus, although the production of long male signals may be associated with higher energy costs, as well as increased risk of competition and predation [109], they may be more effective for the transmission of intraspecific information.

## 7. Multimodal Signalling

Although most research has focused on single-signal systems, it is unlikely that mate finding and selection is always based on a single modality. Moreover, different habitats are likely to promote the use of one type of signal over another, because vibrational and chemical signals have different transmission properties. Indeed, in several vibrational studies, not all mating events were preceded by vibrational signalling, suggesting that other signals may have been used [59,63]. Likewise, rapid wing vibrations have been observed in the olfactometer during chemical signalling studies [100]. Several studies report observing antennation and rapid wing vibration during mate finding and courtship [67,68,70]. Clearer evidence of multimodal signalling systems in Psylloidea was provided by Wenninger *et al.* [78], since males of *D. citri* were shown to increase their calling rate in the absence of female odours. Such a modulation of vibrational signals in response to the presence or absence of female odorants has also been reported in other hemipterans, e.g., *Nezara viridula* [37]. The varying role and interdependence of signal modalities remain under-investigated. More studies are needed to investigate how widespread multimodal signalling is within Psylloidea and whether the operation of these mating strategies is influenced by the presence of conspecific or heterospecific individuals.

## 8. Future Directions

Future studies of signals mediating mate finding and courtship should consider the possibility that some psyllid species may utilize a polyandrous mating system in which elevated levels of male-male competition may result from longer female refractory periods between matings. Both males and females may initiate signalling, but more work is needed to understand the frequency of female initiated signalling. To minimise contamination associated with solvent extraction and the loss of smaller molecules, analytical techniques for capturing volatiles (including headspace collection using solid-phase micro-extraction (SPME)) or direct sampling of weakly or non-volatile compounds by direct injection of whole insects into a GC should be considered. Surface cuticular extracts should be collected by rubbing of the cuticle with SPME fibres and/or silica-rubbing [112]). GC linked Electroantennogram Detection (EAD) or Single-Sensillum Recording (SSR)studies should be used to validate the physiological activity of putative sex attractants. To elucidate the existence of contact chemoreception, behavioural bioassays in appropriately-sized arenas should be used to compare male responses to freshly killed females, with *versus* without cuticular hydrocarbons [113,114,115]. The possible role played by visual cues has been almost entirely ignored. Studies by Burts *et al.* [62] and Wenninger *et al.* [56] have both suggested the possible use of visual cues by *C. pyricola* and *D. citri*, respectively, during mate finding. Species, such as *Casuarinicola australis* (Triozidae) and *Casuarinicola warrigalensis* (Triozidae), exhibit sexual dimorphism of wing patterns (maculation) [116], raising the possibility that they could provide mate finding and perhaps courtship cues.

Since the Psylloidea are so rich in species, exhibit unpredictable fluctuations in abundance and are so economically important, it is vital that we better understand the mechanisms that facilitate aggregation and enhance their reproductive success. The global importance of some psyllid species has already begun this process, but we need to extend it to those psyllid species waiting for us to alter conditions in their native habitats or to relocate them and their hosts overseas.
