# Correction: Lubanga, U.K., *et al.* Semiochemical and Vibrational Cues and Signals Mediating Mate Finding and Courtship in Psylloidea (Hemiptera): A Synthesis. *Insects* 2014, 5*,* 577–595

**DOI:** 10.3390/insects6030743

**Published:** 2015-08-20

**Authors:** Umar K. Lubanga, Christelle Guédot, Diana M. Percy, Martin J. Steinbauer

**Affiliations:** 1 Department of Ecology, Environment & Evolution, La Trobe University, Melbourne, VIC 3086, Australia; E-Mail: M.Steinbauer@latrobe.edu.au; 2 Department of Entomology, University of Wisconsin, Madison, WI 53706, USA; E-Mail: guedot@wisc.edu; 3 Natural History Museum, London SW7 5BD, UK; E-Mail: d.percy@nhm.ac.uk

The authors wish to make the following corrections to this paper [1]:

(1) Figure 3A, presented on page 586, shows a recording that at the time of publication was believed to be a duet between a male and female *Aacanthocnema dobsoni* (Hemiptera: Triozidae). This recording was made on a branchlet supporting a pair of psyllids which is why we believed it to be a duet. Recent recordings from isolated males are similar to recordings known to be duets. We now know that male calls comprise syllables of varying length and structure (including long-syllables and the short-syllables) while female calls comprise short-syllables only. The short-syllables produced by males are similar in structure to female syllables. We are convinced that Figure 3A in the original article does not represent a duet but rather a male call. Consequently we wish to make the following alterations to Figure 3A which we hereby label 3A (i):

Replace the term duet with ♂ call,

Remove the ♂ and ♀ symbols,

Remove the term reply latency (which applies only to duets).

In this correction, we show a typical male phrase Figure 3A (ii) and a typical female call Figure 3A (iii).

Kindly replace:


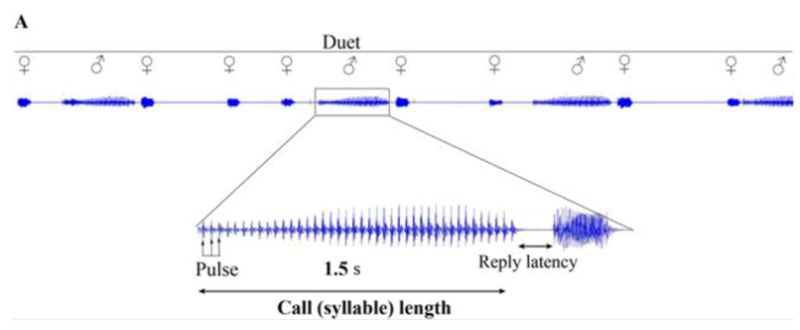


with


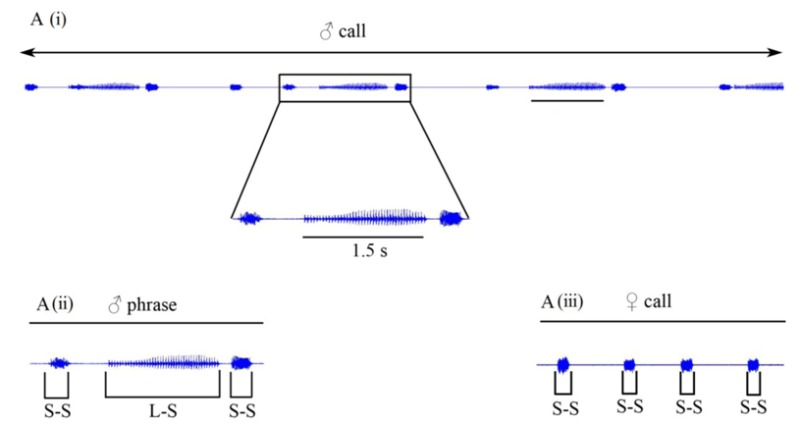


(2) Change to Figure 3 legend. Following the alterations listed above, the Figure 3 legend should be changed as follows:

From:

Vibrational duetting in triozid psyllids. (**A**) *Aacanthocnema dobsoni*; long, simple male call (syllable) and short female reply (syllable). (**B**) *Schedotrioza apicobystra* (published with permission from CSIRO publishing) short and complex, tightly synchronized male-female duet. s = seconds.

To:

Vibrational signalling in triozid psyllids. (**A**) (i) *Aacanthocnema dobsoni*, male call comprising multiple phrases; A (ii) typical male phrase comprising two short- and one long-syllable; A (iii) four short syllables comprising a female call. (**B**) Vibrational duet in *Schedotrioza apicobystra* (published with permission from CSIRO publishing) short and complex, tightly synchronized male-female duet. s = seconds, L-S = long syllable, S-S = short syllable.

The authors would like to apologize for any inconvenience caused to the readers by these changes.
